# Association of cardiorespiratory fitness with adverse outcomes in patients with and without atrial fibrillation: a prospective cohort study

**DOI:** 10.7150/ijms.110802

**Published:** 2025-06-09

**Authors:** Yutong Wang, Tao Xu, Chenxi Xia, Xinyang Song, Yanwen Chen, Sixian Weng, Fang Wang

**Affiliations:** 1 Cardiology department, Beijing Hospital, National Center of Gerontology, Institute of Geriatric Medicine, Chinese Academy of Medical Sciences & Peking Union Medical College, Beijing 100005, China.; 2 Cardiology Department, Beijing Hospital, National Center of Gerontology, Beijing 100005, China.; 3 Peking University Fifth School of Clinical Medicine, Beijing 100005, China.; 4 Department of Cardiology, Beijing Anzhen Hospital, Affiliated to Capital Medical University, Beijing 100005, China.

## Abstract

**Background:** Cardiorespiratory fitness plays a crucial role in cardiovascular health; however, its effects on adverse cardiovascular outcomes across different diseases remain poorly defined. Specifically, the differential impact of cardiorespiratory fitness on patients with and without atrial fibrillation (AF) is yet to be fully understood. This study aimed to explore the relationships between resting heart rate (RHR), maximal heart rate (HRmax), and maximal oxygen uptake (VO₂max) in relation to adverse cardiovascular outcomes, providing valuable insights to inform exercise prescriptions and cardiac rehabilitation practices.

**Methods:** Participants were classified into two groups: those with AF diagnosed prior to baseline (AF group) and those without AF at baseline (non-AF group). In the AF group, outcomes included heart failure (HF), stroke, and all-cause mortality; in the non-AF group, incident AF, stroke, HF, and mortality were assessed. Associations between cardiorespiratory indices—RHR, HRmax, and VO₂max—and adverse cardiovascular events were evaluated using Cox proportional hazards models. Dose-response relationships were examined via restricted cubic spline (RCS) models with three knots.

**Results:** In the non-AF population, higher resting heart rate was significantly associated with an increased risk of adverse cardiovascular outcomes, including heart failure (HF: HR = 1.008, 95% CI 1.001-1.014, P = 0.0182), stroke (HR = 1.010, 95% CI 1.004-1.016, P = 0.0018), atrial fibrillation (AF: HR = 1.011, 95% CI 1.007-1.015, P < 0.0001), and all-cause mortality (HR = 1.016, 95% CI 1.010-1.022, P < 0.0001). In contrast, higher HRmax was inversely associated with these outcomes (HF: HR = 0.993, 95% CI 0.991-0.995, P < 0.0001; stroke: HR = 0.993, 95% CI 0.990-0.995, P < 0.0001; AF: HR = 0.993, 95% CI 0.991-0.994, P < 0.0001; cardiovascular death: HR = 0.994, 95% CI 0.990-0.997, P < 0.0001). Similarly, higher cardiorespiratory fitness, measured by VO₂max, was consistently associated with reduced risks of adverse outcomes (HR range: 0.930-0.961, P < 0.001).

In the AF population, higher RHR was associated with elevated risks of HF (HR = 1.007, 95% CI 1.002-1.012, P = 0.0047) and all-cause mortality (HR = 1.009, 95% CI 1.004-1.014, P < 0.0001). Conversely, greater VO₂max was linked to reduced risks of adverse outcomes, including HF (HR = 0.934, 95% CI 0.899-0.972, P < 0.0001), stroke (HR = 0.943, 95% CI 0.891-0.999, P = 0.0446), and all-cause mortality (HR = 0.957, 95% CI 0.918-0.998, P = 0.038).

**Conclusion:** In individuals without AF, higher resting heart rate was significantly associated with increased risks of incident AF, HF, stroke, and all-cause mortality, with the lowest risks of AF and HF observed at an RHR of 61 beats per minute. Among patients with AF, elevated RHR was significantly linked to higher risks of HF and all-cause mortality. Conversely, higher VO₂max was consistently associated with reduced risks of adverse outcomes across both populations. Furthermore, VO₂max showed strong predictive value for adverse cardiovascular prognostic risks in individuals with and without AF.

## Introduction

In modern society, as economies develop, there is increasing emphasis on healthy lifestyles and their potential benefits, with particular focus on physical activity 1-3. Studies have demonstrated the effects of physical activity on diseases and their outcomes 4, 5. Given the variations in intensity and volume, physical activity can exert differential effects on health outcomes 6, 7. Since individuals can adjust their physical activity levels based on personal capacity, this study focuses on individuals' capacity for physical activity—namely, cardiorespiratory fitness 8, 9—rather than just the quantity or intensity of physical activity or exercise.

Cardiorespiratory fitness reflects aerobic capacity and circulatory efficiency in delivering oxygen throughout the body, serving as a key indicator of fitness and overall health 10-14. The gold standard of cardiorespiratory fitness is maximal oxygen uptake (VO₂max), typically assessed via cardiopulmonary exercise testing (CPET) 15. However, due to the inconvenience and difficulty of CPET, it is not a routine clinical examination. As a result, VO₂max is often estimated using predictive formulas 16, 17. Despite its importance, cardiorespiratory fitness levels have declined globally, largely due to increased sedentary behavior, rising obesity rates, socioeconomic changes, and decreasing physical activity 18-20.

Alongside VO₂max, heart rate metrics such as resting heart rate (RHR) and maximum heart rate (HRmax) are important indicators of cardiorespiratory fitness. RHR, regulated by the autonomic nervous system, is a predictor of mortality and prognosis in many cardiovascular diseases, as well as the occurrence of cardiovascular disease in healthy individuals 21-24. HRmax is the highest heart rate achieved during maximal exercise intensity, beyond which oxygen consumption and heart rate cannot increase further. A decrease in HRmax indicates a decline in cardiac pacemaker function and is an independent risk factor associated with aging 25.

While studies have shown associations between cardiorespiratory fitness and cardiovascular diseases, including AF, few have explored the relationship between HRmax and cardiovascular disease 26-28. To address this gap, this study evaluates the impact of VO_2_max, RHR, and HRmax on cardiovascular disease by comparing populations with and without AF and assessing their prognoses. These findings aim to provide exercise and physical activity guidance for patients with atrial fibrillation and offer scientific insights into exercise to maintain cardiovascular health in the general population, reinforcing the importance of cardiorespiratory fitness.

## Methods

### Data Source and Study Participants

This study utilized data from the UK Biobank, a large-scale biomedical resource comprising comprehensive genetic, clinical, and health information from 502244 participants in the UK, aged 37 to 73 years. Baseline data were collected between 2006 and 2010 at 22 assessment centers across the UK, with ongoing follow-up assessments. Ethical approval for the UK Biobank was granted by the North West Multi-Centre Research Ethics Committee (reference 11/NW/0382), and all participants provided written informed consent.

This study included two population groups: the AF group and the non-AF group. In the non-AF group, we excluded individuals who (1) had withdrawn consent for participation in the UK Biobank (n=6), (2) lacked of exposure or covariates data (n= 413228), or (3) had a history of atrial fibrillation, heart failure, or stroke at baseline, or were deceased (n=17866). A total of 71,144 participants were included in this group. The AF group consisted of individuals diagnosed with atrial fibrillation at baseline (n=5664). Exclusion criteria included individuals who (1) had withdrawn consent for participation in the UK Biobank(n=0), (2) lacked exposure or covariates data (n=3849), or (3) had a history of heart failure or stroke at baseline, or were deceased(n=1166). A total of 649 participants were included in this group.

### Assessment of exposure

The submaximal bicycle ergometer test was conducted using a stationary eBike equipped with Firmware v1.7. Participants were assigned individualized exercise protocols, and electrocardiograms (ECGs) were recorded (CAM-USB 6.5; Cardiosoft software version 6.51) across four distinct phases: the pretest phase (15 seconds), the constant phase (2 minutes at a workload of 30 W for women and 40 W for men), the incremental phase (4 minutes with workload progression to 35% of the maximum estimated workload for low-risk participants and 50% for minimal-risk participants), and the recovery phase (1 minute).

Maximum workload was estimated using a predictive model incorporating age and physical examination findings. It is important to note that in the UK Biobank, ergometry is restricted to a maximum of 50% of the intended maximum workload. Additionally, the linear relationship between heart rate and workload may not persist up to maximum heart rate, particularly in older individuals 29. Resting heart rate was measured by ECG during a period of quiet rest. Estimated VO₂max was calculated using a previously validated method 16, 17, with data expressed in metabolic equivalents (METs), where 1 MET corresponds to 3.5 mL/kg/min 30.

### Assessment of outcomes

Outcomes for atrial fibrillation (AF), heart failure (HF), and stroke were identified using the three-digit International Classification of Diseases, Tenth Edition (ICD-10) codes, a validated method for disease classification and differentiation in the UK Biobank. Mortality outcomes included all-cause death and cardiovascular death. Follow-up began at the date of recruitment and ended at the earliest occurrence of the first disease diagnosis, death, loss to follow-up, or censoring date.

### Covariates

This study assessed multiple covariates, including sociodemographic characteristics, lifestyle factors, physical measurements, and medical history. Sociodemographic characteristics included age, sex (male or female), ethnicity (white or non-white), education level (whether attended college) and the Townsend deprivation index (TDI). Lifestyle factors included smoking status (current, former, or never), alcohol consumption (current, former, or never), and diet quality. Diet quality was assessed using previously reported diet scores, categorized as healthy, moderate, or unhealthy, based on intake of fruits, vegetables, red or processed meats, and salt 31. Physical measurements included blood pressure, waist-to-hip ratio (WHR), and body mass index (BMI). BMI was measured at baseline using impedance methods. Medical history included parental history of cardiovascular disease and personal history of antihypertensive, lipid-lowering, and glucose-lowering medication use.

### Statistical analysis

Baseline demographic and medical characteristics were stratified by sex and presented in ***Table 1***. Continuous variables were expressed as means with standard deviations, and categorical variables as frequencies with percentages.

Two independent research groups were analyzed in this study. In the non-AF group, we assessed the risk of developing atrial fibrillation. In contrast, the AF group did not have the risk of atrial fibrillation calculated, but instead focused on the risk of other outcomes. In this study, ethnic, education level (college), TDI, diet quality, smoking and alcohol status and family history were treated as categorical variables. Age, WHR, BMI, blood pressure, RHR, HRmax, and VO₂max were considered continuous variables. Longitudinal risk analyses were conducted using Cox proportional hazards models to evaluate the associations of RHR, HRmax, and VO₂max with the risk of incident adverse outcomes. Hazard ratio (HR) and 95% confidence intervals (CI) were reported. The dose-response relationships between these factors and adverse outcomes were assessed using restricted cubic spline (RCS) models with three knots. All analyses were adjusted for the covariates described above.

Statistical significance was defined as a two-sided* P*-value < 0.05. Data analysis and visualization were performed using R software (version 4.2.1). The following R packages were used: data.table, dplyr, survival, rms, ggplot2, and ggpubr.

## Results

***Table 1*** presents the baseline characteristics of the study participants. Over a median follow-up period of 13.39 years, 4226 participants developed AF, and 336 CVD events were recorded among individuals with AF over a median of 13.37 years. The loss to follow-up rate was low, and no significant bias was observed in the study population. Among individuals without AF, the average ages were 56.93 years for men and 56.51 years for women. In the AF population, the average ages were higher, at 62.74 years for men and 62.36 years for women. In the non-AF population, women exhibited higher RHR and HRmax compared to men (P < 0.001); however, their VO₂max values were lower than those of men in both the AF and non-AF groups (P < 0.001).

***Table 2*** summarizes the effects of cardiorespiratory fitness and heart rate on adverse cardiovascular outcomes in both non-AF and AF populations. In the population without AF, a higher RHR was significantly associated with an increased risk of adverse cardiovascular outcomes, including HF (HR = 1.008, 95% CI 1.005-1.012, *P* = 0.0001), cardiovascular death (HR = 1.011, 95% CI 1.005-1.016, *P* < 0.0004) and all-cause death (HR = 1.016, 95% CI 1.014-1.019, *P* < 0.0001), except for stroke (HR = 1.004, 95% CI 0.999-1.009, *P* = 0.1132). In contrast, a higher HRmax was significantly associated with a lower incidence of adverse outcomes, including HF (HR = 0.993, 95% CI 0.991-0.995, *P* < 0.0001), stroke (HR = 0.993, 95% CI 0.990-0.995, *P* < 0.0001), AF (HR = 0.993, 95% CI 0.991-0.994, *P* < 0.0001), and cardiovascular death (HR = 0.994, 95% CI 0.990-0.997, *P* = 0.0005), except for all-cause mortality (HR = 0.999, 95% CI 0.997-1.000, *P* = 0.0693). Moreover, higher VO₂max values were significantly associated with a lower incidence of adverse events (HR range: 0.918-0.968, *P* < 0.001). In the AF population, a higher RHR was significantly associated with an increased risk of adverse outcomes, specifically HF (HR = 1.022, 95% CI 1.010-1.034, *P* = 0.0002) and all-cause death (HR = 1.018, 95% CI 1.005-1.030, *P* = 0.0053), but not with stroke (HR = 1.003, 95% CI 0.984-1.023, *P* = 0.7244) or cardiovascular death (HR = 1.010, 95% CI 0.987-1.033, *P* = 0.3836). Additionally, higher VO₂max was significantly associated with a lower risk of adverse outcomes, including HF (HR = 0.931, 95% CI 0.895-0.968, *P* = 0.0003) and all-cause death (HR = 0.957, 95% CI 0.918-0.997, *P* = 0.0376), except for cardiovascular death (HR = 0.956, 95% CI 0.888-1.030, *P* = 0.236) and stroke (HR = 0.945, 95% CI 0.892-1.002, *P* = 0.0567). In patients with AF, HRmax was not associated with adverse cardiovascular outcomes.

**Figure 1:**
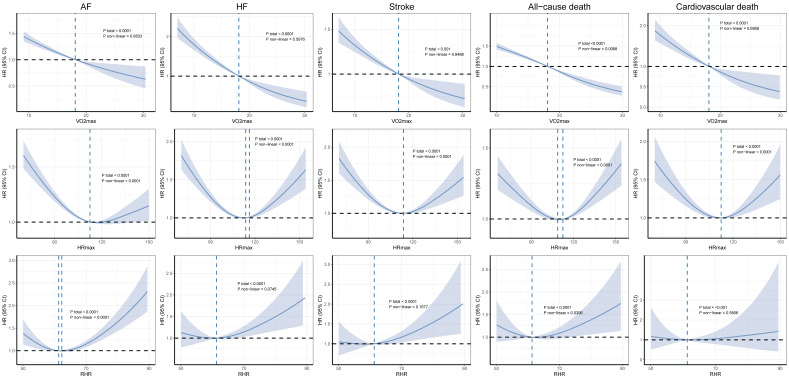
** Dose-response associations between cardiorespiratory fitness and risk of incident HF, AF, stroke, and mortality in participants without AF.** The Cox regression models with restricted cubic splines were used to explore the dose-response relationship between cardiorespiratory fitness and outcomes. The HR and their 95% CI were reported. The Cox proportional hazard models were adjusted for The Cox proportional hazard models were adjusted for age, sex, ethnicity, education, Townsend deprivation index, smoking status, alcohol consumption, diet quality, blood pressure, waist-to-hip ratio, body mass index, parental history of cardiovascular disease, personal history of antihypertensive, lipid-lowering, and glucose-lowering medication use. Abbreviation: HR, hazard ratios, CI, confidence interval, AF, atrial fibrillation, HF, heart failure.

**Figure 2:**
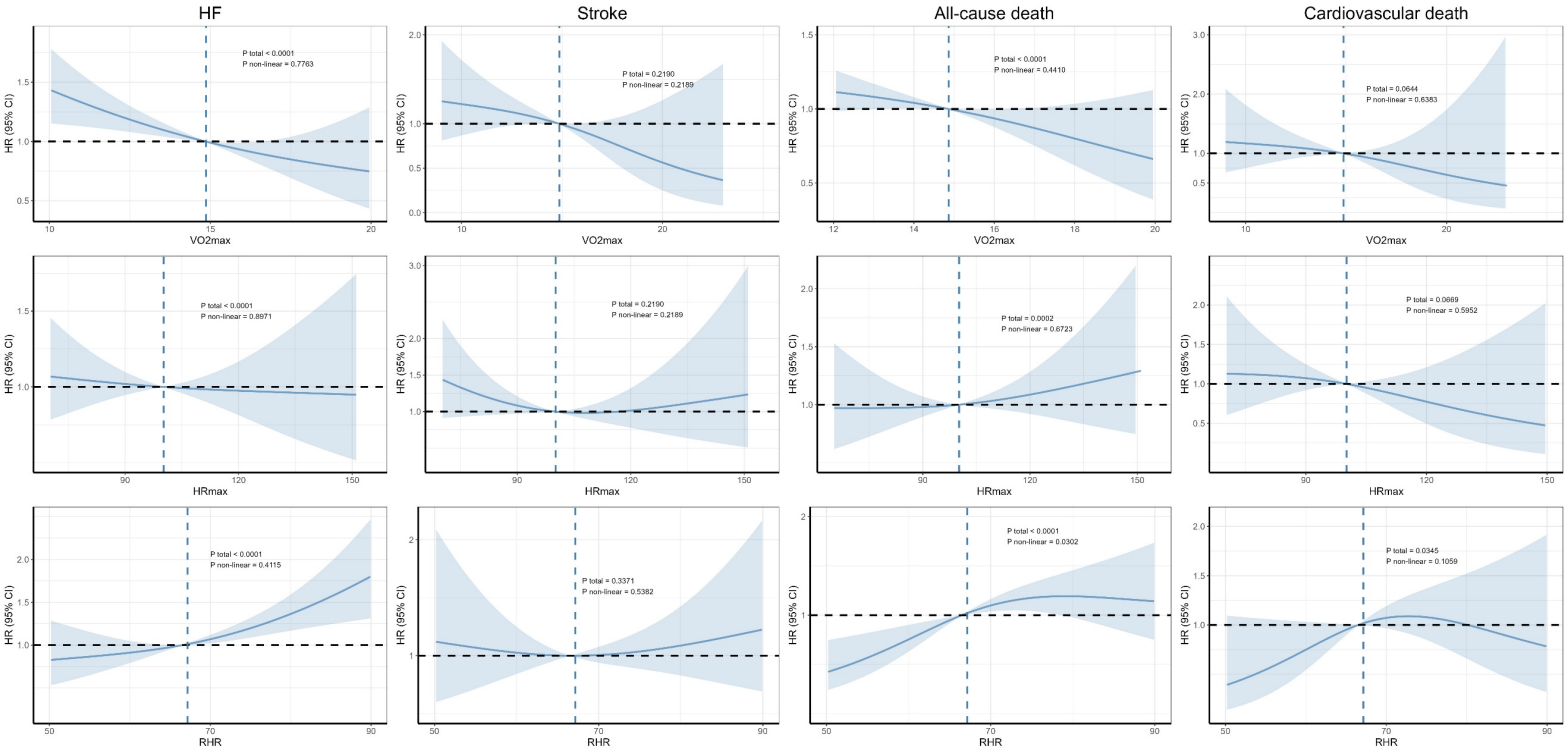
** Dose-response associations between cardiorespiratory fitness and risk of incident HF, AF, stroke, and mortality in participants with AF.** The Cox regression models with restricted cubic splines were used to explore the dose-response relationship between cardiorespiratory fitness and outcomes. The HR and their 95% CI were reported. The Cox proportional hazard models were adjusted for The Cox proportional hazard models were adjusted for age, sex, ethnicity, education, Townsend deprivation index, smoking status, alcohol consumption, diet quality, blood pressure, waist-to-hip ratio, body mass index, parental history of cardiovascular disease, personal history of antihypertensive, lipid-lowering, and glucose-lowering medication use. Abbreviation: HR, hazard ratios, CI, confidence interval, AF, atrial fibrillation, HF, heart failure.

In the non-AF population (***Figure 1***), VO₂max did not exhibit nonlinear relationships with the outcomes, except for all-cause death (*P*non-linear = 0.0088). HRmax showed nonlinear associations with all outcomes, forming U-shaped curves in the RCS plots. Similarly, the relationships between RHR and both AF (*P*non-linear < 0.0001) and all-cause mortality (*P*non-linear = 0.0306) also displayed nonlinear U-shaped curves. In contrast, in the AF population, no significant nonlinear relationships were observed (***Figure 2***).

## Discussion

The main findings of this study are as follows. Firstly, in the population without AF, RHR was significantly associated with the risk of incident AF, HF, cardiovascular death, and all-cause mortality. Patients with an RHR of 61 beats per minute had the lowest risk of AF and HF. Given the gender differences in heart rate observed in the non-AF group, subgroup analysis by sex was performed. In females, an RHR of 60 beats per minute was associated with the lowest risk of HF, while an RHR of 62 beats per minute was linked to the lowest risk of AF. In males, the lowest risk of HF was observed at an RHR of 61 beats per minute, while the lowest risk of AF occurred at an RHR of 62 beats per minute. Additionally, the relationships between HRmax and these outcomes exhibited nonlinear U-shaped curves. Secondly, in the AF population, RHR was significantly associated with the risk of HF and all-cause mortality in a linear manner.

In contrast, HRmax was not associated with adverse cardiovascular outcomes, possibly due to the influence of AF on heart rate. Lastly, a higher VO₂max was significantly associated with a lower risk of developing AF, HF, stroke, and death in the non-AF population. In those already diagnosed with AF, a higher VO₂max was significantly associated with a lower risk of HF, stroke, and all-cause mortality. The relationships between VO₂max and adverse cardiovascular outcomes were all linear, demonstrating that VO₂max has significant predictive ability for adverse cardiovascular prognostic risks in both populations.

Resting heart rate, a simple and easily measurable indicator, is medically predictive of many adverse outcomes 32-34. Previous studies have confirmed that RHR predicts adverse outcomes in patients with diabetes and aortic disease 35, 36. Additionally, RHR appears to have predictive ability for the incidence of cardiovascular diseases such as coronary disease, atrial fibrillation, and heart failure 37-39. Individuals with regular exercise habits and better cardiorespiratory fitness generally have a lower RHR, contributing to better mental health and cardiovascular fitness 40. Furthermore, RHR can also be a predictor of VO₂ max, indicating a potential correlation between them 41. Our study showed that an RHR of 60-62 beats per minute was associated with the lowest likelihood of developing AF and HF, which is consistent with previous studies from Spain 42, where an RHR around 70 had the lowest risk of cardiovascular disease, and from the Asia-Pacific region 43, where an RHR over 75 was associated with an increased risk of premature mortality.

HRmax is an important indicator of cardiorespiratory fitness and serves as the basis for determining the maximum level of cardiovascular fitness and prescribing suitable exercise intensity. HRmax represents inherent capacity and typically does not change in response to other factors, although it generally decreases with age 44. To prescribe efficient and precise exercise intensity, the American College of Sports Medicine (ACSM) provides guidelines based on an individual's HRmax, such as exercising at 50-85% of HRmax to improve aerobic fitness 45. In our study, an association was found between HRmax and adverse cardiovascular outcomes. Cox analysis showed that higher achievable HRmax was associated with a lower risk of adverse outcomes in the general population. Since HRmax reflects cardiorespiratory well-being, this underscores the importance of cardiorespiratory fitness for cardiovascular protection.

For patients with permanent or persistent AF, controlling the ventricular rate is a crucial treatment strategy, except in most cases of paroxysmal AF. Ventricular rate control through medication or ablation has been shown to improve symptoms, quality of life, and exercise capacity 46. Medications such as beta-blockers used to control the ventricular rate reduce the incidence of poor prognosis and mortality 47. In our study, RHR was associated with the risk of HF and all-cause mortality in patients with AF, whereas HRmax did not show a significant association. The relationship between heart rate and AF compared to normal heart rhythm remains unclear. In patients with AF, RHR may be related to increased sympathetic tone and decreased parasympathetic activity. Dysfunction of the cardiac autonomic nervous system, particularly heightened sympathetic activity, significantly contributes to the development of various cardiovascular conditions—including diabetes, myocardial infarction, and stroke—and is linked to negative health outcomes 48, 49. Heart rate variability (HRV) during normal heart rhythms serves as an indicator of stroke risk; however, in patients with AF, HRV measurements are unreliable 50. This unreliability may explain why no significant relationship was observed between HRmax and adverse outcomes in individuals with AF. Heart rate may be influenced by irregularities in the atrioventricular node and subnodal conduction pathways, not just by parasympathetic activity as in normal rhythm. Structural issues in these areas could lead to abnormal heart function and indicate poorer long-term health outcomes 51. Some studies have shown that heart rate is not associated with poor prognosis in AF, although controlling the ventricular rate still has positive implications 52. Furthermore, RHR and HRmax is related to heart rate reserve, which has also been associated with poor prognosis in patients with AF in recent years 53.

Although heart rate parameters, such as RHR, are widely discussed in the medical and health fields, measuring VO₂max through CPET has not become a routine clinical practice in most regions worldwide. This is primarily due to the complexity of the equipment and the testing procedure, which limits the broader clinical application of cardiorespiratory fitness 54. However, with increasing health awareness and the development of sports cardiology and exercise science, the idea of guiding exercise and cardiac rehabilitation through scientific principles has gained popularity 55, 56. While heart rate is susceptible to the symptoms of AF, VO₂ max remains independent of these fluctuations. Patients with higher VO₂ max levels have a lower risk of adverse outcomes and mortality 57, and there may also be a genetic component involved 58. Better cardiorespiratory health significantly reduces the risk of CVD, emphasizing the need to focus more on cardiorespiratory fitness as a marker of CVD risk, especially in middle-aged and older populations 59. In our study, VO₂ max was a clear predictor of a lower risk of adverse cardiovascular outcomes in both populations with and without AF, demonstrating a significant linear relationship. Unlike simply calculating the volume or intensity of exercise, VO₂ max is an inherent property that does not change easily but improves as one's exercise capacity increases.

Currently, CPET is not a routine measurement, and few individuals voluntarily undergo CPET to determine their VO₂ max. Based on our results, people should be encouraged not only to do exercise and physical activity regularly but also to engage in exercise and physical activity scientifically, using appropriate tests to guide their activities.

## Limitation

Firstly, instead of directly measuring VO₂ max, this study estimated it using a formula, which may introduce some inaccuracies. However, the data used for the calculations—such as heart rate, age, and weight—were directly measured instead of estimated, enhancing the credibility and reliability of the results. Secondly, since AF can affect heart rate, further studies in patients with other cardiovascular diseases are needed in the future. Additionally, there are several limitations related to the study population that need to be addressed: 1. The majority of the study population consisted of individuals of European descent, which may not fully represent global populations. Future studies should include more diverse ethnic groups to provide a broader perspective. 2. The study population had a higher proportion of middle-aged and elderly individuals, which led to a skewed distribution of data, such as heart rate, potentially introducing bias. 3. The data used in this study was collected early from 2006 to 2010, and given the changes in global health trends over time, more recent data will be necessary to validate and extend these findings. These considerations highlight areas for future research to enhance the generalizability and relevance of the results.

## Conclusion

In conclusion, RHR was significantly associated with the risk of AF, HF, cardiovascular death, and all-cause mortality. In females, an RHR of 60 beats per minute was associated with the lowest risk of HF, while an RHR of 62 beats per minute was linked to the lowest risk of AF. In males, the lowest risk of HF was observed at an RHR of 61 beats per minute, while the lowest risk of AF occurred at an RHR of 62 beats per minute. In patients with AF, RHR was significantly associated with the risk of HF and all-cause mortality, and a higher VO₂ max was associated with a lower risk of adverse outcomes. VO₂ max proved to be a significant predictor of adverse cardiovascular prognosis in both populations with and without AF.

## Figures and Tables

**Table 1:** Baseline characteristics

Characteristics	Population without AF			Population with AF		
	Female	Male	*P*	Female	Male	*P*
Sample size, n	39283	31861		194	455	
Age, years	56.51 (8.00)	56.93 (8.23)	< 0.001	62.36 (5.34)	62.74 (5.84)	0.4141
Ethnic, white, n (%)	35706 (90.89)	29120 (91.40)	0.01976	186 (95.88)	431 (94.73)	0.673
College, n (%)	13537 (34.46)	11862 (37.23)	< 0.001	57 (29.38)	129 (28.35)	0.8644
WHR	0.82 (0.07)	0.94 (0.07)	< 0.001	0.84 (0.08)	0.95 (0.07)	< 0.001
BMI, kg/m2	26.94 (5.10)	27.77 (4.17)	< 0.001	29.32 (5.86)	28.95 (4.66)	0.4265
SBP, mmHg	134.82 (19.00)	139.99 (17.11)	< 0.001	138.77 (19.91)	138.75 (18.34)	0.9909
DBP, mmHg	80.36 (9.89)	83.52 (9.79)	< 0.001	80.53 (11.32)	82.06 (11.08)	0.1132
TDI, n (%)			0.02748			0.5588
Low	14589 (37.14)	11662 (36.60)		72 (37.11)	179 (39.34)	
Intermediate	13327 (33.93)	10687 (33.54)		72 (37.11)	149 (32.75)	
High	11367 (28.94)	9512 (29.85)		50 (25.77)	127 (27.91)	
Diet quality, n (%)			< 0.001			0.015
Healthy	3213 (8.18)	1168 (3.67)		21 (10.82)	23 (5.05)	
Intermediate	31261 (79.58)	23406 (73.46)		142 (73.20)	337 (74.07)	
Unhealthy	4809 (12.24)	7287 (22.87)		31 (15.98)	95 (20.88)	
Smoking status, n (%)			< 0.001			0.06351
Never	23824 (60.65)	16022 (50.29)		94 (48.45)	177 (38.90)	
Previous	12401 (31.57)	12179 (38.23)		84 (43.30)	241 (52.97)	
Current	3058 (7.78)	3660 (11.49)		16 (8.25)	37 (8.13)	
Alcohol consumption, n (%)			< 0.001			0.3985
Never	2384 (6.07)	942 (2.96)		8 (4.12)	11 (2.42)	
Previous	1348 (3.43)	1055 (3.31)		14 (7.22)	27 (5.93)	
Current	35551 (90.50)	29864 (93.73)		172 (88.66)	417 (91.65)	
History of CVD, n (%)	10817 (27.54)	10921 (34.28)	< 0.001	50 (25.77)	125 (27.47)	0.7264
RHR, beats per minute	62.80 (10.40)	60.97 (10.86)	< 0.001	61.00 (7.75)	62.93 (10.66)	0.4798
HRmax, beats per minute	112.57 (20.43)	108.61 (18.42)	< 0.001	98.07 (26.00)	101.72 (24.58	0.09668
VO2max, ml/ (kg·min)	16.22 (4.38)	18.68 (4.86)	< 0.001	12.26 (4.35)	14.89 (5.00)	< 0.001

Continuity and categorical variables are shown as mean (SD) and number (percentage), respectively. Abbreviation: BMI, body mass index; WHR, waist-to-hip ratio; TDI, townsend deprivation index; SBP: systolic blood pressure; DBP: diastolic blood pressure; RHR: resting heart rate; HR: heart rate; VO_2_max: maximal oxygen consumption.

**Table 2:** Hazard Ratios for All the Outcomes

Outcomes	Patients without AF						Patients with AF					
	Resting heart rate		Maximum heart rate		VO_2_max		Resting heart rate		Maximum heart rate		VO_2_max	
	*HR (95%CI)*	*P value*	*HR (95%CI)*	*P value*	*HR (95%CI)*	*P value*	*HR (95%CI)*	*P value*	*HR (95%CI)*	*P value*	*HR (95%CI)*	*P value*
HF	1.008 (1.005-1.012)	< 0.0001	0.993 (0.991-0.995)	< 0.001	0.931 (0.922-0.940)	< 0.001	1.022 (1.010-1.034)	0.0002	0.998 (0.992-1.005)	0.6183	0.931 (0.895-0.968)	0.0003
Stroke	1.004 (0.999-1.009)	0.1132	0.993 (0.990-0.995)	< 0.001	0.941 (0.930-0.952)	< 0.001	1.003 (0.984-1.023)	0.7244	0.995 (0.984-1.006)	0.346	0.945 (0.892-1.002)	0.0567
AF	0.993 (0.990-0.996)	< 0.001	0.993 (0.991-0.994)	< 0.001	0.961 (0.954-0.968)	< 0.001	/	/	/	/	/	/
Allcause Death	1.016 (1.014-1.019)	< 0.0001	0.999 (0.997-1.000)	0.0693	0.951 (0.945-0.957)	< 0.001	1.018 (1.005-1.030)	0.0053	1.003 (0.996-1.010)	0.3838	0.957 (0.918-0.997)	0.0376
Cardiovascular Death	1.011 (1.005-1.016)	0.0004	0.994 (0.990-0.997)	0.0005	0.932 (0.918-0.946)	< 0.001	1.010 (0.987-1.033)	0.3836	0.992 (0.978-1.005)	0.2309	0.956 (0.888-1.030)	0.236

The Cox proportional hazard models were adjusted for age, sex, ethnicity, education, townsend deprivation index, smoking status, alcohol consumption, diet quality, blood pressure, waist-to-hip ratio, body mass index, parental history of cardiovascular disease, personal history of antihypertensive, lipid-lowering, and glucose-lowering medication use. Abbreviation: AF, atrial fibrillation; HF, heart failure; VO_2_max: maximal oxygen consumption.

## Abbreviations

CRF: cardiopulmonary fitness
VO2max: maximal oxygen uptake
RHR: resting heart rate
HRmax: maximal heart rate
AF: atrial fibrillation
HF: heart failure
CPET: cardiopulmonary exercise testing
